# Curcumin Induces Autophagy-mediated Ferroptosis by Targeting the PI3K/AKT/mTOR Signaling Pathway in Gastric Cancer

**DOI:** 10.5152/tjg.2024.23526

**Published:** 2024-08-01

**Authors:** Xin Zheng, Jun Liu, Wei Hu, Bin Jiang, Xin Zhou, Min Zhang, Ming Song

**Affiliations:** 1 Department of General Surgery, Wuhan Third Hospital, Wuhan, Hubei, China; 2 Department of Neurosurgery, Wuhan Third Hospital, Wuhan, Hubei, China

**Keywords:** Gastric cancer, curcumin, ferroptosis, autophagy, PI3K/AKT/mTOR

## Abstract

**Background/Aims:**

As a very common malignancy of the digestive system, the incidence and mortality rates of gastric cancer (GC) are increasing year by year. The critical role of ferroptosis in cancer development has been well-documented. The polyphenol compound curcumin shows prominent anti-tumor effects in multiple cancer types, including GC. However, whether curcumin participates in GC tumorigenesis by regulating ferroptosis remains unknown.

**Materials and Methods:**

Gastric cancer cells AGS and HGC-27 were treated with curcumin (0, 10, and 20 μM). Cell viability and death were evaluated through CCK-8 and LDH release assays. LC3B expression in cells was estimated through immunofluorescence staining. Intracellular ferrous iron (Fe^2+^), GSH, MDA, and lipid ROS levels were assessed by corresponding assay kits. The cellular levels of autophagy markers (ATG5, ATG7, Beclin 1, and LC3B), ferroptosis markers (ACSL4, SLC7A11, and GPX4), and phosphorylated (p)-PI3K, p-AKT, and p-mTOR were determined through western blotting.

**Results:**

Curcumin attenuated cell viability but stimulated cell death in GC cells. Curcumin enhanced autophagy in GC cells, as demonstrated by the increased levels of ATG5, ATG7, Beclin 1, and LC3B. Besides, curcumin upregulated iron, MDA, GSH, and ACSL4 levels while downregulated lipid ROS, SLC7A11, and GPX4 levels, suggesting its stimulation on ferroptosis in GC cells. Curcumin decreased p-PI3K, p-AKT, and p-mTOR levels in cells. Importantly, the ferroptosis inhibitor ferrostatin-1 overturned the impacts of curcumin on GC cell viability, death, and ferroptosis.

**Conclusion:**

Curcumin suppresses GC development by inducing autophagy-mediated ferroptosis by inactivating the PI3K/AKT/mTOR signaling.

**Main Points**

- Curcumin inhibits cell viability but promotes cell apoptosis in gastric cancer (GC) cells.
- Curcumin induces autophagy in GC cells.
- Curcumin promotes ferroptosis in GC cells.
- Curcumin suppresses the PI3K/AKT/mTOR pathway in GC cells.
- Curcumin inhibits cell viability but promotes cell apoptosis in GC cells by inducing ferroptosis.

## Introduction

Globally, gastric cancer (GC) ranks fifth in terms of prevalence and fourth regarding cancer-related death.^1^ Currently, surgical resection is the primary means of clinical treatment for GC, with chemotherapy as the main adjunctive approach. Platinum-based chemotherapeutic agents are highly effective broad-spectrum anticancer drugs and the first-line therapeutic drugs for GC patients, particularly those in advanced stages.^2^ However, chemotherapeutic drugs can also damage normal cells, impairing the digestive system, nervous system, and cardiovascular system, and causing patients to suffer from adverse symptoms such as nausea, vomiting, and hair loss. Therefore, it is urgent to develop safer and more efficient drugs. Numerous natural products have received much attention since they exhibit potential therapeutic effects on cancers with fewer side effects on normal cells.^3^

**Figure 1.**
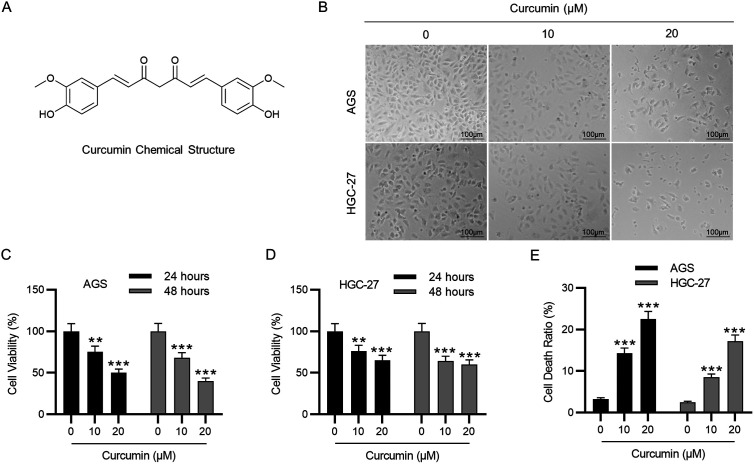
Curcumin inhibits cell viability but promotes cell apoptosis in GC cells. (A) The chemical structure of curcumin. (B) Observation of GC cell morphology and counts after curcumin (0, 10, 20 μM) treatment under a microscope. Scale bars = 100 μm. (C,D) Detection of GC cell viability after curcumin (0, 10, 20 μM) treatment for 24 and 48 hours through CCK-8 assay. (E) Measurement of GC cell death following curcumin (0, 10, 20 μM) treatment by LDH release assay. **P < .01, ***P < .001.

Curcumin (chemical structure shown in Figure 1A) is a yellow polyphenol compound separated from the root of the *Curcuma longa* (turmeric) plant.^4^ Curcumin has been confirmed to possess anti-oxidant, anticancer, anti-inflammatory, anti-analgesic, lipid-modifier, and anti-microbial effects.^5^ Ferroptosis is a form of cell death driven by iron-dependent lipid peroxidation.^6^ Increasingly studies have revealed that inducing ferroptosis can kill tumor cells and repress tumor growth, making it an effective cancer treatment strategy.^7^ Curcumin has previously been reported to suppress tumorigenesis in many types of human cancers by facilitating ferroptosis.^8-10^ Even though numerous studies have demonstrated that curcumin exerts potent anti-tumor effects in the progression of GC,^11^ whether curcumin mediates GC development through regulating ferroptosis remains unclear.

We aimed to explore the functions of curcumin in regulating ferroptosis in GC and the underlying mechanism. Our results suggested that curcumin repressed GC cell viability but promoted GC cell death through enhancing ferroptosis. Moreover, curcumin-induced autophagy inactivated the PI3K/AKT/mTOR signaling in GC cells, which might explain the molecular mechanisms of curcumin in regulating GC development.

## Materials and Methods

### Cell Culture and Treatment

As reported in previous studies,^12,13^ 2 human GC cells (AGS and HGC-27) acquired from ATCC (Manassas, VA, USA) were used in our study. Both cells were grown in DMEM (Gibco, Gaithersburg, MD, USA) containing 10% fetal bovine serum (FBS, Gibco) and 1% penicillin–streptomycin (Gibco) at 37°C in 5% CO_2_. Curcumin (HY-N0005; purity: 98.16%; MedChem Express, Monmouth Junction, NJ, USA) was dissolved in 0.1% DMSO to obtain a 50 mM stock solution and was further diluted in a cell culture medium until specific concentrations (0, 10, and 20 μM) for the subsequent cell treatment for 24 or 48 hours. To confirm the role of ferroptosis in curcumin-mediated GC cell viability and death, ferrostatin-1 (HY-100579; purity: 99.96%; MedChem Express), a selective inhibitor of ferroptosis, was also dissolved in DMSO and used for the combined treatment of cells with curcumin (20 μM) and ferrostatin-1 (50 nM) for 24 hours.

### CCK-8 Assay

Gastric cancer cell viability in response to curcumin treatment alone or combined treatment with curcumin and ferrostain-1 was assessed using the Cell Counting Kit-8 (Beyotime). Briefly, cells were seeded (1 × 10^4^/mL, 100 μL/well) onto 96-well plates. After the indicated treatment for the indicated times, CCK-8 solution (10 μL) was added to each well, and then cells were incubated for another 2 hours. Finally, the OD value at 450 nm was detected using a microplate reader (BioRad, Richmond, CA, USA).

### LDH Release Assay

The LDH Cytotoxicity Assay Kit (Roche, Basel, Switzerland) was adopted to determine the cell death rate. Briefly, 1 × 10^4^ AGS and HGC-27 cells were seeded into 96-well plates and received the indicated treatment. Subsequently, the cell culture supernatant was collected, and LDH release in the supernatant was measured using the LDH Cytotoxicity Assay Kit. The absorbance value was read at 490 nm with a microplate reader.

### Detection of Iron, GSH, MDA, and Lipid ROS Levels

The levels of intracellular ferrous iron (Fe^2+^), GSH, malondialdehyde (MDA), and lipid reactive oxygen species (ROS) in GC cells following curcumin treatment alone or combined treatment with curcumin and ferrostatin-1 were measured using the Iron Assay Kit (MAK025; Sigma-Aldrich), Lipid Peroxidation MDA Assay Kit (S0131S; Beyotime), GSH Assay Kit (S0053; Beyotime), and ROS Assay Kit (S0033S; Beyotime). The absorbance was measured at 593 nm, 532 nm, 412 nm, and 525 nm, respectively, using a spectrophotometer.

### Western Blotting

Western blotting was performed to analyze the protein levels in cell cultures under different conditions and treatments. Briefly, total cellular protein was extracted using RIPA lysis buffer (Servicebio, Wuhan, China) containing protease and phosphatase inhibitors, followed by quantification of the extracted protein concentration using a BCA Protein Assay Kit (Servicebio). Protein (50 µg per lane) was loaded into 10% SDS-PAGE gels and then transferred to polyvinylidene fluoride membranes. After being blocked with 5% skim milk powder for 1 hour, the membranes were incubated overnight at 4°C with primary antibodies (Abcam, Cambridge, UK) and for 1 hour at 25°C with the HRP‑conjugated secondary antibody (ab205718, 1/2000, Abcam). The primary antibodies used were: ATG5 (ab109490, 1/1000), ATG7 (ab133528, 1/10 000), Beclin 1 (ab207612, 1/2000), LC3B (ab192890, 1/2000), ACSL4 (ab205199, 1/1000), SLC7A11 (ab175186, 1/1000), GPX4 (ab125066, 1/1000), p-PI3K (ab182651, 1/500), PI3K (ab133595, 1/1000), p-AKT (ab183758, 1/1000), AKT (ab200195, 1/2000), p-mTOR (ab109268, 1/1000), mTOR (ab134903, 1/10 000), and GAPDH (ab128915, 1/10 000). GAPDH served as the loading control. Finally, enhanced chemiluminescence reagents were used to visualize the target protein bands, whose grayscale was analyzed using ImageJ software.

### Immunofluorescence Staining

Immunofluorescence staining was conducted in GC cells to determine the expression of LC3B. After the indicated treatment, AGS and HGC-27 cells were washed 3 times with prechilled PBS, fixed with 4% paraformaldehyde for 15 minutes, immersed in 0.1% Triton X-100 for 10 minutes, and then blocked with 3% bovine serum albumin for 30 minutes. Afterward, cells were incubated overnight at 4°C with an anti-LC3B primary antibody (ab51520, 1/2000, Abcam), followed by another 1 hour incubation in the dark with Alexa Fluor 647-labeled secondary antibody (ab150083, 1/1000, Abcam). After nuclear staining with 4’,6-diamidino-2-phenylindole (Roche) for 15 minutes, the slides were sealed with an anti-fluorescence quenching sealing agent and imaged by a fluorescence microscope (DM2500, Leica).

### Statistical Analysis

The results from at least 3 independent experiments were analyzed using GraphPad Prism software (version 6.0, San Diego, CA) and are expressed as the mean ± SD. Student’s *t*-test and one-way ANOVA followed by Bonferroni’s post-hoc test were used to compare the differences between 2 groups and among multiple groups, respectively. *P* < .05 was considered statistically significant.

## Results

### Curcumin Attenuates Cell Viability and Enhances Cell Apoptosis in GC Cells

First, GC cells following curcumin treatment were observed under microscopy to examine their morphology and counts, through which we found that curcumin treatment resulted in remarkable cell death (Figure 1B). Then, the CCK-8 assay showed that GC cell growth was evidently attenuated after curcumin treatment (Figure 1C-D). Furthermore, the LDH release assay revealed that the cell death ratio in curcumin-treated GC cells was notably higher than in control GC cells (Figure 1E). Importantly, curcumin (20 μM) exerted better anti-growth and pro-death effects on GC cells than curcumin (10 μM).

### Curcumin Induces Autophagy in GC Cells

**Figure 2.**
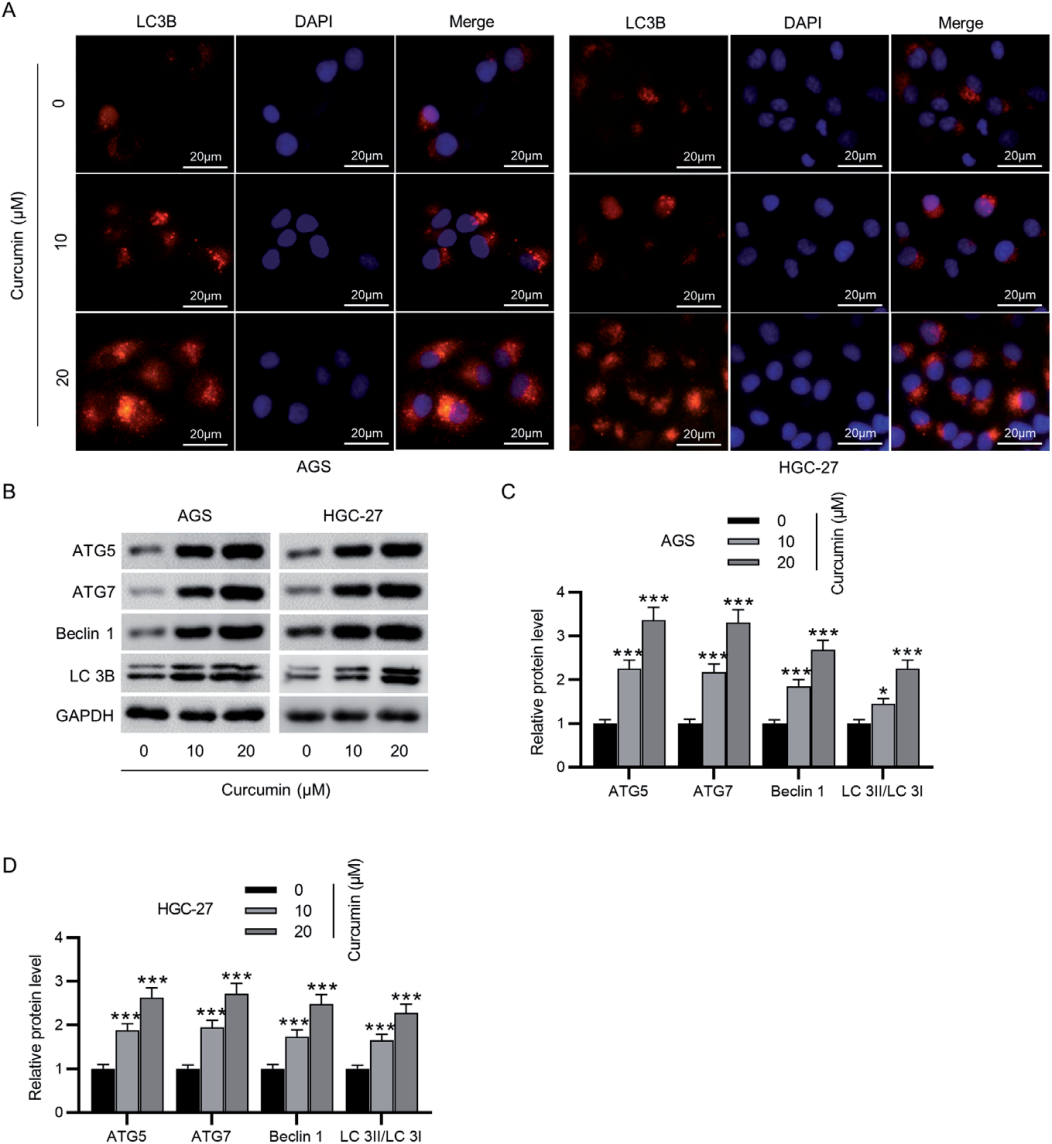
Curcumin induces autophagy in GC cells. (A) Assessment of LC3B expression in curcumin (0, 10, 20 μM)-treated GC cells through immunofluorescence staining. Scale bars = 20 μm. (B–D) Measurement of ATG5, ATG7, Beclin 1, and LC3B protein levels in curcumin (0, 10, 20 μM)-treated GC cells by western blotting. *P < .05, ***P < .001.

Next, we evaluated the influence of curcumin on autophagy markers in GC cells. According to the immunofluorescence staining of LC3B, curcumin-treated GC cells exhibited markedly stronger immunofluorescence intensity than untreated GC cells (Figure 2A), indicating that LC3B expression was enhanced in GC cells after curcumin treatment. Moreover, western blotting illustrated that curcumin treatment led to an obvious increment in ATG5, ATG7, Beclin 1, and LC3B protein levels (Figure 2B-D). Taken together, curcumin treatment induces autophagy in GC cells.

### Curcumin Promotes Ferroptosis in GC Cells

**Figure 3.**
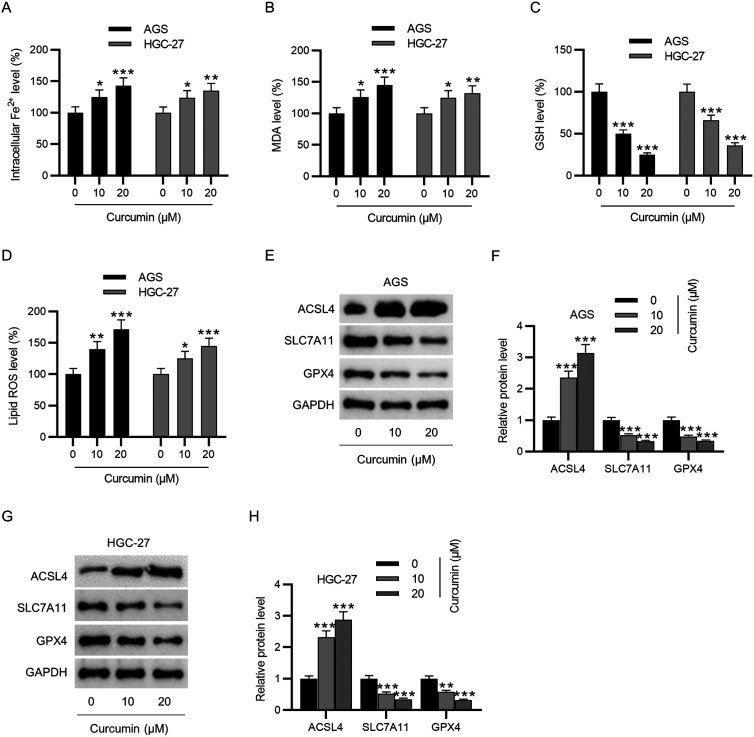
Curcumin promotes ferroptosis in GC cells. (A–D) Measurement of iron, MDA, GSH, and lipid ROS levels in GC cells after curcumin (0, 10, 20 μM) treatment using corresponding assay kits. (E–H) Determination of ACSL4, SLC7A11, and GPX4 protein levels in GC cells after curcumin (0, 10, 20 μM) treatment through western blotting. *P < .05, **P < .01, ***P < .001.

To determine whether curcumin influences ferroptosis in GC cells, the intracellular ferrous iron level after curcumin treatment was assayed. As revealed in Figure 3A, curcumin treatment considerably elevated the levels of iron in GC cells. Malondialdehyde and ROS levels were substantially elevated while GSH level was reduced by curcumin treatment (Figure 3B-D), indicating that curcumin suppressed the accumulation of ROS and lipid peroxide generated by iron metabolism in GC cells. Additionally, the levels of ferroptosis-associated proteins were evaluated through western blotting, from which we discovered that curcumin treatment enhanced ACSL4 protein levels but weakened SLC7A11 and GPX4 protein levels in GC cells dose-dependently (Figure 3E-H). Overall, curcumin treatment facilitates ferroptosis in GC cells.

### Curcumin Suppresses the PI3K/AKT/mTOR Signaling

**Figure 4.**
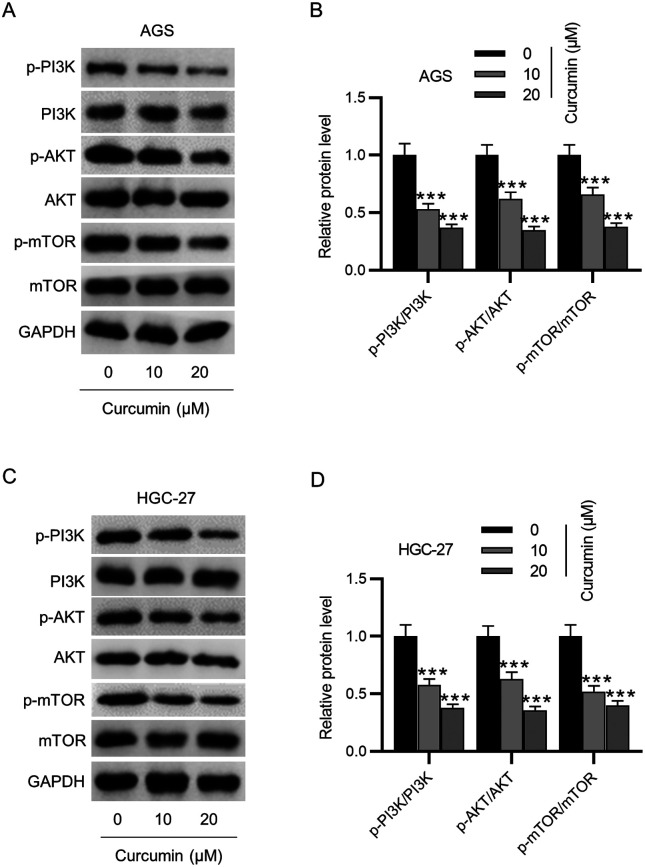
Curcumin suppresses the PI3K/AKT/mTOR pathway in GC cells. (A–D) Determination of phosphorylated (p)-PI3K, p-AKT, and p-mTOR protein levels in GC cells after curcumin (0, 10, 20 μM) treatment via western blotting. ***P < .001.

Since curcumin was reported to modulate the PI3K/AKT/mTOR signaling, which is closely related to autophagy and ferroptosis, we next tested whether curcumin affects the PI3K/AKT/mTOR signaling in GC cells. As shown by western blotting, p-PI3K, p-AKT, and p-mTOR levels in GC cells notably declined following curcumin treatment (Figure 4A-D). Accordingly, we speculated that curcumin might induce autophagy and ferroptosis in GC cells by restraining the PI3K/AKT/mTOR signaling.

### The Ferroptosis Inhibitor Ferrostatin-1 Reverses the Influence of Curcumin on GC Cell Viability and Death

**Figure 5.**
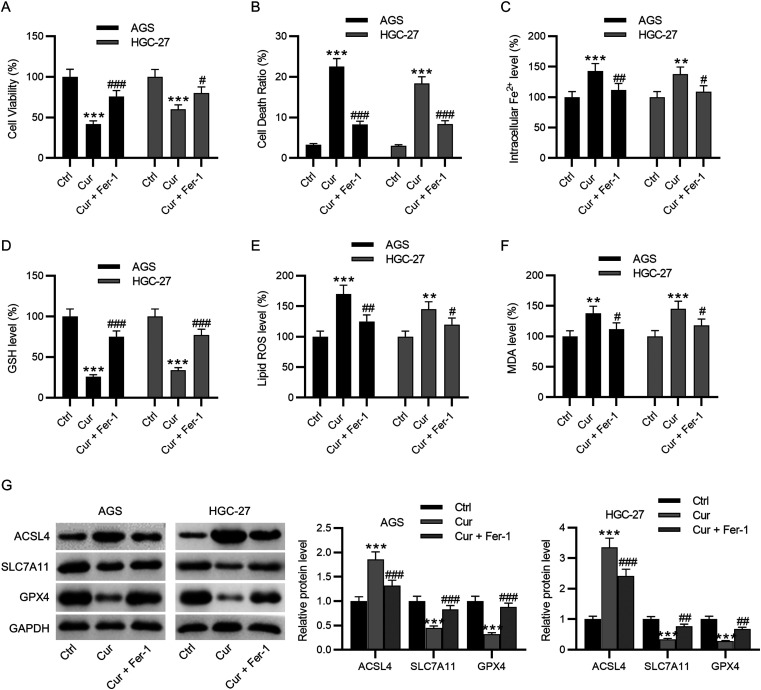
The ferroptosis inhibitor ferrostatin-1 reverses the impacts of curcumin on GC cell viability and death. (A) Evaluation of GC cell viability after treatment with curcumin (20 μM) combined with or without the ferroptosis inhibitor ferrostatin-1 via CCK-8 assay. (B) Detection of GC cell death following the above treatment by LDH release assay. (C–F) Estimation of iron, MDA, GSH, and lipid ROS levels in GC cells after the above treatment by using corresponding assay kits. (G) Measurement of ACSL4, SLC7A11, and GPX4 protein levels in GC cells following the above treatment through western blotting. **P < .01, ***P < .001 vs. Ctrl; #*P* < 0.05, ##*P* < 0.01, ###*P* < 0.001 vs. Cur.

Finally, curcumin-treated GC cells were further treated with ferrostatin-1 to assess the influence of ferroptosis suppression on curcumin-induced changes on GC cell viability and death. The results demonstrated that the curcumin-induced decline in cell viability and enhancement in cell death were reversed by ferrostatin-1 treatment (Figure 5A-B). In addition, ferrostatin-1 treatment overturned curcumin-induced increment in iron, ROS, and MDA levels and decrement in GSH level in GC cells, indicating that curcumin-induced abnormal iron deposition and oxidative stress were ameliorated after treatment with the ferroptosis inhibitor (Figure 5C-F). Western blotting manifested that the promotion of curcumin on ACSL4 protein levels and its inhibition on SLC7A11 and GPX4 protein levels were debilitated by ferrostatin-1 treatment (Figure 5G). Collectively, GC curcumin represses GC cell viability but promotes GC cell death by enhancing ferroptosis.

## Discussion

Numerous studies have revealed that active compounds in natural herbal plants can be prepared as potential therapeutic agents for GC due to their low toxicity and mild effects. It has been demonstrated that high concentrations of plant-derived natural compounds have excellent biological activities in suppressing tumor growth and inducing tumor cell apoptosis. Curcumin is an acidic polyphenolic compound whose anti-tumor functions and potential regulatory mechanisms have been identified in multiple types of human cancers, including GC. Our research elucidated that curcumin treatment reduced GC cell growth but enhanced cell death. Besides, curcumin induced ferroptosis and autophagy and inactivated PI3K/AKT/mTOR signaling in GC cells. Importantly, the ferroptosis inhibitor ferrostatin-1 overturned the impacts of curcumin on GC cell viability and death. Accordingly, curcumin exerted an antitumorigenic effect on GC by promoting ferroptosis.

Ferroptosis is a unique form of cell death first proposed in 2012, which is distinct from other forms of cell death morphologically, biochemically, and genetically. The essence of ferroptosis is that the failure of the membrane lipid repair enzyme GPX4 leads to the impairment in the metabolism of intracellular lipid oxides, which, catalyzed by iron ions, results in ROS accumulation on membrane lipids, eventually causing an intracellular redox imbalance and triggering cell death. Tumor cells have a higher demand for iron than normal cells, which makes tumor cells more susceptible to iron death when iron levels are increased.^14^ Therefore, ferroptosis can increase the mortality of tumor cells and overcome the drug resistance of tumor cells through synergistic effects with other cell death pathways, thus becoming a new cancer therapeutic target.^15^ Ferroptosis plays a vital role in modulating tumor growth, including GC, hepatocellular carcinoma, lung cancer, and renal cell carcinoma.^16^ A previous study showed that the levels of ferroptosis-related targets NRF2, GPX4, and SLC7A11 were markedly upregulated in GC tissues and cells versus normal controls.^17^ Several natural-plant-derived compounds have been proven to suppress GC progression by inducing ferroptosis.^18,19^

Previously, curcumin and its analogs were demonstrated to hinder the development of various cancers through the induction of ferroptosis. For instance, Chen et al clarified that curcumin impeded tumorigenesis in follicular thyroid cancer by activating the ferroptosis pathway by increasing the expression of HO-1.^9^ Zhou et al^20^ disclosed that curcumin suppressed the GSH-GPX4 and FSP1-CoQ10-NADH pathways to induce ferroptosis, thereby attenuating the self-renewal potential of lung cancer stem cells. Shi et al^21^ found that NL01, the derivative of curcumin, not only induced ferroptosis in ovarian cancer cells through reducing HCAR1/MCT1 expression and activating the AMPK/SREBP1 pathway in vitro but also promoted iron death and repressed ovarian cancer growth in xenograft mouse models. Chen et al^22^ suggested that treatment with ALZ003, a curcumin analog, reduced ROS accumulation, lipid peroxidation, and GPX4 expression in glioblastoma cells, suggesting the presence of ALZ003-induced ferroptosis. Even though existing studies have elucidated that curcumin participates in GC progression,^11^ the role of curcumin in regulating GC cell ferroptosis remains unknown. Herein, we found that curcumin treatment upregulated intracellular ferrous iron (Fe^2+^), MDA, lipid ROS, and ACSL4 levels but downregulated GSH, SLC7A11, and GPX4 levels in GC cells, confirming that curcumin-induced ferroptosis in GC cells. Additionally, the ferroptosis inhibitor ferrostatin-1 antagonized the inhibition of curcumin on GC cell viability and its promotion of cell death, validating that curcumin retarded GC growth and development by inducing ferroptosis.

Autophagy is an intrinsic homeostatic mechanism for eukaryotic cells to maintain homeostasis in the internal environment. Autophagy plays a key role in many pathological processes in human diseases, including neurological diseases, tumors, cardiovascular diseases, infections, metabolic diseases, and autoimmune diseases. The autophagy pathway has also become a potential target therapeutic pathway for many diseases.^23^ Autophagy can both promote and suppress tumor.^24^ Furthermore, autophagy has been discovered to stimulate ferroptosis through multiple mechanisms. It can destroy iron-stored protein ferritin heavy chain, allowing the accumulation of iron, which breaks cellular iron homeostasis and results in ferroptosis.^25^ Many studies have shown that induction of autophagy-mediated ferroptosis contributes to hindering tumor development.^26^ Previously, the promotion of curcumin on autophagy in cancer cells has been illustrated. For example, curcumin represses cell viability and induces apoptosis and protective autophagy in GC cells.^27^ Curcumin also restrains cell viability but enhances renal cell carcinoma apoptosis and autophagy by suppressing the AKT/mTOR pathway.^28^ Curcumin inhibits cell proliferation and tumor growth but facilitates cell death in lung cancer by inducing ferroptosis through activating autophagy.^29^ Additionally, the previous study suggested that curcumin stimulated autophagy by repressing the PI3K/AKT/mTOR pathway,^30^ which is one significant signaling pathway involved in cancer development. Our results showed that curcumin increased ATG5, ATG7, Beclin 1, and LC3B protein levels but decreased phosphorylated (p)-PI3K, p-AKT, and p-mTOR levels in GC cells, indicating that curcumin-induced autophagy but inhibited PI3K/AKT/mTOR signaling.

To be honest, while our paper elucidates the specific role of curcumin in regulating ferroptosis in GC cells and predicts the possible molecular mechanism, there also exist certain limitations. First, the induction of ferroptosis and autophagy by curcumin and its repression on PI3K/AKT/mTOR signaling were not certified *in vivo*. Second, our study only verified that curcumin regulated GC cell growth and death by promoting ferroptosis. The definite relationship among ferroptosis, autophagy, and PI3K/AKT/mTOR signaling in mediating GC development was not validated. These deficiencies need to be addressed in further research by using experimental animal models and the PI3K agonist.

In summary, our research confirms that curcumin suppressed cell growth but promotes cell death in GC cells by stimulating ferroptosis. Mechanistically, curcumin induces autophagy and inactivates the PI3K/AKT/mTOR pathway in GC cells. Hence, curcumin might suppress GC development by inducing autophagy-mediated ferroptosis by restraining the PI3K/AKT/mTOR signaling. Even though many existing literatures have respectively confirmed the role of curcumin and ferroptosis in GC, their relationships in GC have not been elucidated before. Accordingly, our observations might expand our knowledge of the effects of curcumin on ferroptosis in GC and provide a novel direction for the potential use of curcumin in GC treatment.

## Data Availability Statement

The datasets used or analyzed during the current study are available from the corresponding author on reasonable request.
